# The Effect of Vitamin D Supplementation on Clinical and Laboratory Biomarkers in Patients with Rheumatoid Arthritis: A Systematic Review and Meta-Analysis of Randomised Controlled Trials

**DOI:** 10.31138/mjr.090725.lar

**Published:** 2025-09-30

**Authors:** Zahra Khatirnamani, Karimollah Hajian-Tilaki, Behzad Heidari, Ahmad Sohrabi

**Affiliations:** 1 Student Research Centre, Research Institute, Babol University of Medical Sciences, Babol, Iran;; 2 Department of Biostatistics and Epidemiology, School of Public Health, Babol University of Medical Sciences, Babol, Iran;; 3 Social Determinants Health Research Centre, Research Institute, Babol University of Medical Sciences, Babol, Iran;; 4 Department of Internal Medicine, Division of Rheumatology, Ayatollah Rohani Hospital, Babol University of Medical Sciences, Babol, Iran;; 5 Cancer Control Research Centre, Cancer Control Foundation, Iran University of Medical Sciences, Tehran, Iran

**Keywords:** Vitamin D, rheumatoid arthritis, C-reactive protein, serum vitamin D, visual analogue scale

## Abstract

**Aim:**

Rheumatoid arthritis (RA) is recognised as an inflammatory condition. Evidence indicates that vitamin D modulates inflammation by influencing diverse immune cells. The purpose of this systematic review and meta-analysis was to assess the effectiveness of vitamin D as a supplement for RA in comparison to a control group.

**Methods:**

We searched PubMed, Embase, Scopus, and Web of Science databases through March 2025 using targeted search terms. Our inclusion criteria encompassed clinical studies that enrolled RA patients and compared vitamin D supplementation against either placebo or standard care protocols. The results from the chosen studies were reported as Standardised mean differences (SMD) using random effect model with a 95% confidence interval.

**Results:**

Vitamin D supplementation resulted in a significant improvement in the VAS [SMD = −1.54, 95% CI (−2.53, −0.55), P = 0.002], serum vitamin D level [SMD = 1.52, 95% CI (0.86, 2.17), P = 0.001] and CRP [SMD = −0.88, 95% CI (−1.31, −0.44), P =0.001] but not in other outcomes. There was considerable heterogeneity among the studies for VAS (I^2^ = 97.4%, P = 0.001), DAS28 (I^2^ = 89.1%, P = 0.001), serum vitamin D Levels (I^2^ = 94.7%, P = 0.001), CRP (I^2^ = 84.1%, P = 0.001), and ESR (I^2^ = 84.1%, P = 0.001).

**Conclusion:**

The intervention groups receiving vitamin D supplementation showed statistically significant improvements in serum vitamin D levels, visual analog scale VAS scores, and CRP levels compared to control groups among RA patients.

## INTRODUCTION

Rheumatoid arthritis (RA) is a chronic autoimmune disease characterised by systemic inflammation, primarily affecting the joints and leading to pain, swelling, stiffness, and, over time, joint destruction. RA progression is driven by immune dysregulation, particularly an imbalance in T-helper (Th) cell subtypes, with Th17 cells playing a central role. Th17 cells produce pro-inflammatory cytokines such as interleukin-17 (IL-17), which sustain inflammation and contribute to tissue damage in RA.^1^

Vitamin D, traditionally recognised for its essential role in calcium and bone metabolism, also plays a crucial part in immune system regulation and exhibits anti-inflammatory properties. The active form of vitamin D, 1,25-dihydroxyvitamin D (1,25(OH)2D), is synthesised primarily in immune cells such as dendritic cells and macrophages. This active form helps modulate immune responses by suppressing the production of Th17-related cytokines, which are known to play a significant role in autoimmune diseases.^2^

There is a strong association between vitamin D deficiency and RA, as shown in a meta-analysis of 15 studies involving 1,143 RA patients and 963 healthy controls. This study found that lower serum vitamin D levels were associated with higher disease activity, as measured by the Disease Activity Score 28 (DAS28), indicating that lower serum vitamin D in RA patients may contribute to the severity of RA.^3^ This link is further supported by another study that found a strong correlation between serum interleukin-17 (IL-17) levels and RA activity, with higher IL-17 levels corresponding to more active disease. Regarding the inverse relationship between serum vitamin D levels and IL-17 activity in patients with RA, raising serum vitamin D levels through supplementation could potentially modulate immune responses and reduce disease activity in RA patients.^4^

These findings provide a rationale for further studies to explore the beneficial effects of vitamin D supplementation. This issue has been examined in several studies. For instance, one study involving 150 RA patients on stable disease-modifying antirheumatic drugs revealed that, in RA patients with low serum vitamin D levels and high disease activity, raising serum vitamin D levels from 10.05 ng/ml to 57.21 ng/mL significantly improved disease activity.^5^ Furthermore, multiple studies have reported that vitamin D supplementation in RA patients improves pain, disease activity, and overall health.^6–8^

However, the influence of vitamin D on disease activity, pain, and inflammatory parameters remains inconsistent across studies. While some studies indicate significant improvements in disease activity and pain, others have reported minimal or no effects on pain or inflammation biomarkers.^9–13^ For example, in one systematic review and meta-analysis of 11 studies, vitamin D supplementation was found to significantly increase serum vitamin D levels and reduce DAS28, C-reactive protein (CRP), and erythrocyte sedimentation rate (ESR), but no significant effects were observed on the Health Assessment Questionnaire (HAQ) or Visual Analog Scale (VAS) for pain.^13^ Another meta-analysis, including 438 participants across six studies, found that vitamin D supplementation improved DAS28, ESR, and tender joint count (TJC) but had no significant impact on CRP or parathyroid hormone (PTH). Notably, the effects varied by ethnic group, with improvements in pain and vitamin D levels seen in European participants, while TJC and vitamin D levels improved in Asian subgroups. Longer supplementation durations (>12 weeks) and higher doses (>50,000 IU) were associated with more significant effects.^12^

Despite these positive findings, substantial heterogeneity regarding baseline vitamin D levels, age, sun exposure, and concurrent medications influencing outcomes has been observed across studies.^11^ This variability highlights the need for a more comprehensive review to evaluate the reliability of vitamin D supplementation in reducing inflammation and improving clinical outcomes in RA. Some studies have examined the effect of vitamin D supplements on RA patients, but they have limitations, as they do not include all eligible papers^12^, and did not specify sources of heterogeneity^11^, or have only examined a subset of information across studies.^13^ Given these inconsistencies, the present systematic review and meta-analysis aims to synthesise the available evidence from randomised controlled trials (RCTs) to assess the effects of vitamin D supplementation on clinical and laboratory components RA activity by consolidating the findings. This study seeks to provide clearer insights into the potential therapeutic role of vitamin D in RA management.

## METHODS

### Research Strategy

We searched the PubMed, Embase, Scopus and Web of Science databases for studies published before March 2025. The search terms were (1) “Vitamin D”[Mesh] OR “Ergocalciferols”[Mesh] OR “Cholecalciferol”[Mesh] OR “Calcifediol”[Mesh] OR “Ergocalciferol” OR “Vitamin D Supplementation” OR “25-hydroxy-vitamin D”; (2) “Arthritis”[Mesh] OR “Rheumatoid Arthritis”[Mesh] OR “Rheumatoid Nodule” OR “Rheumatoid Vasculitis”; and (3) #1 AND #2. The above search strategy was run in PubMed and was tailored to each database when necessary (**Table S1**, **Appendix**). Two independent reviewers selected and screened all results, and in cases of disagreement, a third reviewer was asked for advice. The reviewers applied the PRISMA statement guidelines for reporting systematic reviews and meta-analyses.^14^

### Eligibility Criteria

The inclusion criteria for this systematic review were as follows: (1) a RCT design related to the therapeutic effect of vitamin D as a supplement was used; (2) human subjects were recruited; (3) vitamin D supplementation was the main intervention in the experimental group and was compared to a standard treatment control condition; and (4) at least one detail of the outcomes of interest was reported, including changes in serum vitamin D level, ESR, VAS, DAS28, CRP. Data including the mean and standard deviation of each group at baseline and post-intervention, along with the number of participants in each group, were able to be obtained. The exclusion criteria were as follows: (1) duplicate publications; (2) non-intervention designs (such as case-control studies, cohort studies, cross sectional studies, case reports and experiences, theory research, and reviews); and (3) non-clinical tests and animal experiments.

### Data Extraction

Two review authors independently screened the literature using the predetermined inclusion criteria and extracted the data from the trials. The following information was extracted: participant characteristics, intervention and outcome data, adverse effects, and methodological quality. The reviewers resolved any disagreements about the extracted data from the included studies by consensus and consulted a third review author if the disagreements persisted.

### Risk of Bias Assessment

The risk of study bias was assessed using the Cochrane Handbook for Systematic Reviews.^15^ The risk of bias of the studies was evaluated with regard to the following aspects: confounding, measurement of the exposure, selection of participants into the study and other biases.

### Statistical Analysis

The Stata software was used for meta-analysis. Standard deviations (SD) values were used when the data were provided in the same unit. Data conversions were performed when outcome measures were reported in different units. We assessed between-study heterogeneity using Chi-squared and I^2^ statistics, considering P < 0.10 or I^2^ > 50% as thresholds for significant heterogeneity. The random-effect model was used in combing the effect size of interest and the results have been shown by Forest plots and 95% confidence intervals (CIs). Additionally, the subgroup analysis was used to explore the influence of certain characteristics such as geographic regions, duration, supplemental dose and risk of bias. The risk of bias was assessed based on the tools provided by the Cochrane Manual.^15^ Moreover, the funnel plots were depicted and also Eger’s test was performed to assess the possibility of publication bias and p-value<0.05 was considered the presence of publication bias.

### Data Conversion

Missing Standard Deviation Calculations:

For trials reporting average values without SDs, we derived missing standard deviations using two methods:

From Standard Error (SE):

When sample size (n) and SE were available:

$$SD=SE\times \sqrt{n}$$

From 95% Confidence Intervals:

When n, mean, and 95% CI bounds (a=upper, b=lower) were reported:^16,17^

$$\begin{array}{l} SD=a-mean/1.96\sqrt{n}\\ SD=mean-b/1.96\sqrt{n} \end{array}$$

The protocol of this study has been registered with PROSPERO under the number: CRD420251013948.

## RESULTS

### Study Selection

**Figure 1.**
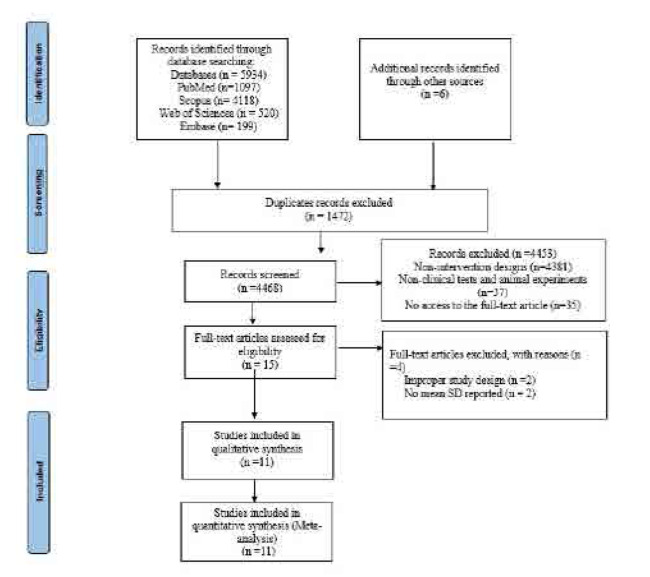
The PRISMA flow diagram.

From an initial pool of 5,934 records, 1,472 duplicates were removed. Title/abstract screening excluded 4,453 additional articles, leaving 15 for full-text assessment. Of these, 38 failed inclusion criteria, 2 lacked appropriate controls, and 2 had unusable data. The final analysis included 11 RCTs, encompassing 1,390 participants. **Figure 1** presents the PRISMA flow diagram illustrating study selection from identification through inclusion.

### Study Features

The analysis incorporated single-centre RCTs published over a 13-year period (2011–2024), encompassing 1,390 participants across studies. Individual trial sample sizes varied substantially, ranging from 11 to 123 participants. Data was collected from studies across multiple continents: Asia,^10,18,19 –22^ North America,^23^ Europe^9,24^ and Africa.^25,26^ Treatment periods ranged between 6 weeks and 6 months, exclusively involving rheumatoid arthritis patients (**Table 1**).

**Table 1.** Summary features of studies and participants.

**First author (Country; year)**	**Sample size (Treatment / Control)**	**Mean age ±SD (Treatment / Control)**	**Dose of vitamin D**	**Duration of intervention**	**Outcome measured**
Gopinath et al. (India, 2011)^18^	(59/62)	(44.86 ±12.33/44.90 ±12.5)	500 IU/Weekly	12 Weeks	VAS
Salesi et al. (Iran, 2012)^10^	(50/48)	(49.9 ± 13.0/50.0 ± 12.7)	50000 IU/Weekly	12 Weeks	VAS, DAS28, Serum Vitamin D, ESR
Hansen et al. (America, 2014)^23^	(11/11)	(63.0 ± 12/53 ± 11.0)	50000 IU/Weekly	8 Weeks	DAS28, Serum Vitamin D
Yang et al. (China, 2015)^19^	(84/88)	(41.2±6.6/41.7±5.8)	140 IU/Weekly	32 Weeks	Serum Vitamin D
Buondonno et al. (Italy, 2017)^9^	(18/18)	(56.0 ± 14.0/54 ± 12.0)	25000 IU/Weekly	12 Weeks	VAS, DAS28, ESR, CRP
Li et al. (1) (China, 2018)^20^	(123/123)	(48.47±11.12/51.12±10.23)	50000 IU/Weekly	6 Weeks	VAS, Serum Vitamin D, ESR, CRP
Li et al. (2) (China, 2018)^20^	(123/123)	(49.58±10.57/51.12±10.23)	50000 IU/Weekly	6 Weeks	VAS, Serum Vitamin D, ESR, CRP
Adami et al. (Italy, 2019)^24^	(26/35)	(58.0±12.0/58.0±13.0)	25000 IU/Weekly	12 Weeks	VAS, DAS28
Mukherjee et al. (India, 2019)^21^	(75/75)	(36.7±10.9/38.8±13.5)	60000 IU/Weekly	8 Weeks	VAS, DAS28
El-Banna et al. (Egypt, 2020)^25^	(40/30)	(45.65±6.92/45.2±3.83)	50000 IU/Weekly	12 Weeks	DAS28
Bansal et al. (India, 2022 )^22^	(50/50)	(41.46±8.89/41.94±9.01)	5600 IU/Weekly	24 Weeks	Serum Vitamin D, ESR, CRP
Elfituri (Libya, 2024)^26^	(48/20)	(47.6 ±11.7/46.8 ±11.3)	50000 IU/Weekly	12 Weeks	DAS28, Serum Vitamin D, CRP, ESR

1 month = 4 Week.

### Assessment of risk of bias

**Figure 2.**
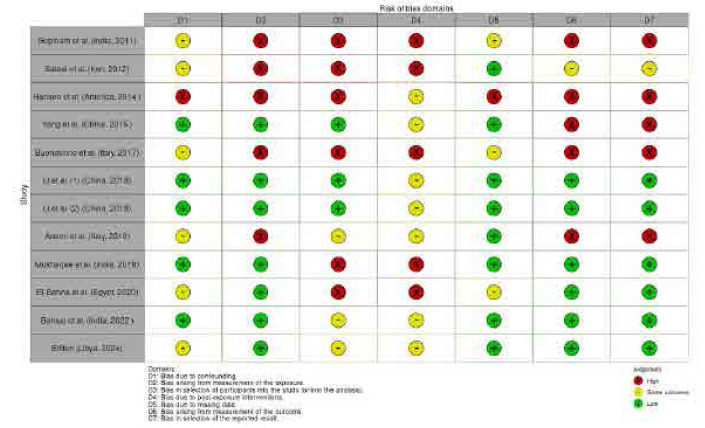
Risk of bias assessment.

The methodological quality and potential biases of the selected studies were visualised in **Figure 2**, following the assessment criteria outlined in the Cochrane Handbook.

### Study Results

**Figure 3.**
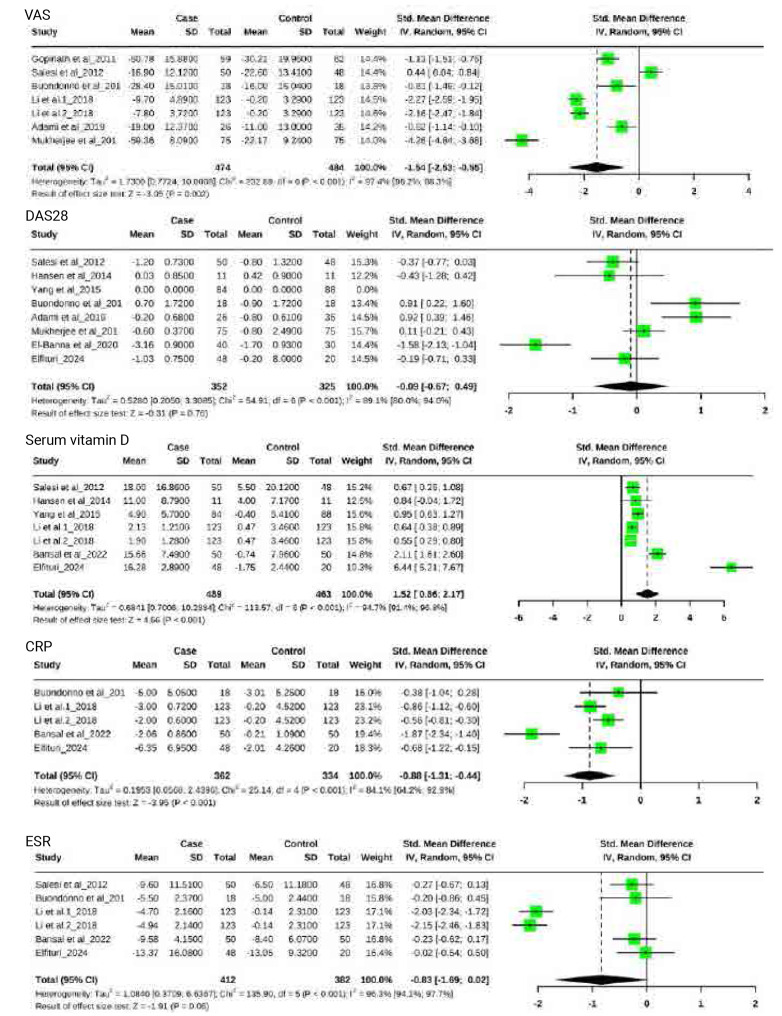
Effect of vitamin D supplementation on outcome measured.

Six included studies of RCT trials with a total of 958 participants provided data on the VAS.^9,10,18,20,21,24^ The VAS in the intervention group was lower than that in the control group, and the difference was significant [SMD = −1.54, 95% CI (−2.53, −0.55), P = 0.002] (Figure 3) with highly significant heterogeneity (I2 = 97.4%, P = 0.001). Vitamin D supplementation produced significant decreases in the following subgroups: Fair, Asia, Europe, duration > 12w, and vitamin D dose ≤ 50,000 IU (**Table 2**).

**Table 2.** Subgroup analysis with respect to study regions, duration and dose of vitamin D and inflammatory factors.

**Subgroups**	**VAS**			**DAS28**			**Serum Vitamin D**			**ESR**			**CRP**		
	**SMD**	**95% CI**	**P**	**SMD**	**95% CI**	**P**	**SMD**	**95% CI**	**P**	**SMD**	**95% CI**	**P**	**SMD**	**95% CI**	**P**
Geographical regions															
North America	-	-	-	−0.43	[−1.28;0.42]	-	0.84	[−0.04;1.72]	-	-	-	-	-	-	-
Europe	−0.69	[−1.10; −0.28]	0.001	0.92	[0.50;1.34]	<0.001	-	-	-	−0.20	[−0.86;0.45]	-	−0.38	[−1.04;0.28]	-
Asia	−1.87	[−3.11; −0.62]	0.003	−0.12	[−0.59;0.36]	0.63	0.95	[0.53;1.37]	<0.001	−1.18	[−2.20; −0.15]	0.02	−1.06	[−1.67; −0.45]	0.32
Africa	-	-	-	−0.88	[−2.25;0.48]	0.020	6.44	[5.21;7.67]	-	−0.02	[−0.54;0.50]	-	−0.68	[−1.22; −0.15]	-
Duration															
≤12w	-	-	-	−0.09	[−0.67;0.49]	0.76	1.56	[0.70;2.41]	<0.001	−0.96	[−1.90;0.01]	0.05	−0.68	[0.87; −0.79]	<0.001
>12 w	-	-	-	-	-	-	1.51	[0.38;2.64]	0.009	−0.23	[−0.62;0.17]	-	−1.87	[−2.34; −1.40]	-
Vitamin D dose															
≤50,000 IU	−1.10	[−1.99; −0.21]	0.02	−0.13	[−0.87,0.61]	0.74	1.52	[0.86;2.17]	<0.001	−0.83	[−1.69;0.02]	0.06	−0.88	[−1.31; −0.44]	-
>50,000 IU	−4.26	[−4.84; −3.68]	-	0.11	[−0.21;0.43]	-	-	-	-	-	-	-	-	-	-
Risk of Bias															
Bad	−1.27	[−2.71;0.17]	0.08	−0.19	[−0.71;0.33]	0.83	0.84	[0.60;1.08]	<0.001	−0.25	[−0.59;0.09]	0.15	−0.38	[−1.04;0.28]	-
Fair	−2.21	[−2.44; −1.99]	<0.001	−0.09	[−0.67;0.49]	-	2.20	[1.03;3.37]	<0.001	−1.12	[−2.18; −0.06]	0.04	−0.97	[−1.46; −0.49]	0.16

DAS28 levels were reported in seven study with a total of 677 participants.^9,10,21,23–26^ There was not a significant difference in the DAS28 between the rheumatoid arthritis patients who received vitamin D supplementation and the control group [SMD = −0.09, 95% CI (−0.67, 0.49), P = 0.76] (**Figure 3**) with high heterogeneity (I2 = 89.1%, P = 0.001). The effects shown in the Europe and Africa subgroups were significantly in favour of the control group (**`**).

Seven studies were included.^10,19,20,22,23,26^ There was a significant increase in the serum vitamin D level in the vitamin D supplementation group [SMD = 1.52, 95% CI (0.86, 2.17), P = 0.001] (Figure 3) with high heterogeneity (I2 = 94.7%, P = 0.001). Similar results were demonstrated in the duration ≤ 12w or >12w, Asia, and vitamin D dose ≤ 50,000 IU and bad or fair Risk of Bias subgroups (**Table 2**).

Four studies involving 696 total participants (221 receiving vitamin D supplementation) demonstrated significantly lower CRP levels in the supplementation group versus controls.^9,20,22,26^ The random-effects model showed a substantial reduction [SMD = −0.88, 95% CI (−1.31, −0.44), P =0.001], though with considerable heterogeneity (I2 = 84.1%, P = 0.001) (**Figure 3**). Similar results were demonstrated in the duration ≤ 12w (**Table 2**).

The meta-analysis of five studies revealed no statistically significant difference in ESR levels between vitamin D supplementation and control groups [SMD = −0.83, 95% CI (−1.69, 0.02), P = 0.06).^9,10,20,22,26^ Notably, we observed substantial heterogeneity across studies (I^2^ = 84.1%, p = 0.001), as illustrated in Figure 3.Similar results have been shown in the duration ≤ 12w, Asia, and vitamin D dose ≤ 50,000 IU and fair Risk of Bias subgroups (**Table 2**).

### Sensitivity analysis

**Supplementary Figure S1.**
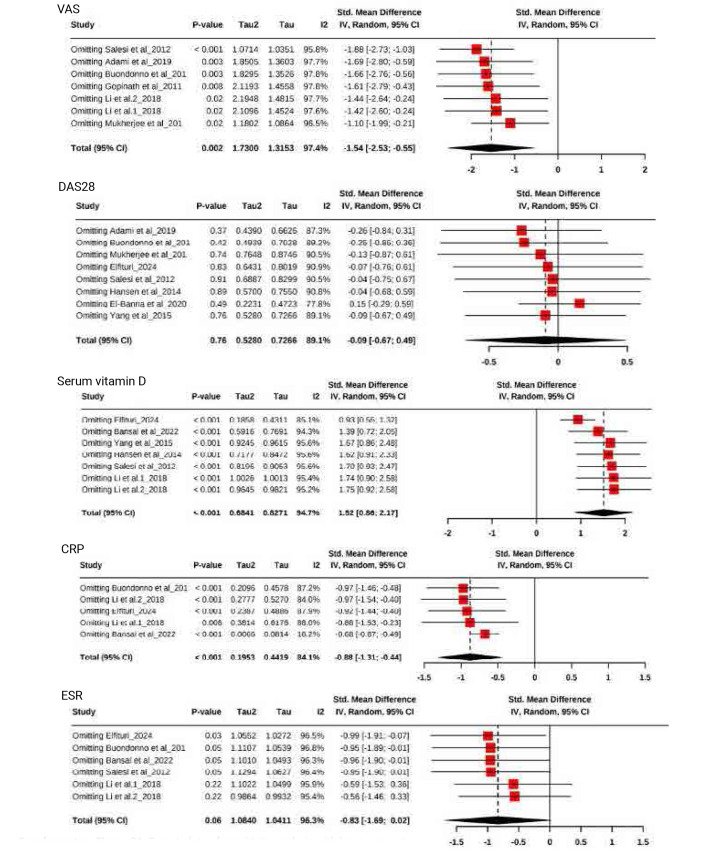
Forest plots of sensitivity analysis with leave-one-out meta-analysis. Results are expressed as Standardised mean difference (SMD) with 95%CI for the remaining studies after excluding one study.

Figure S1 presents the forest plots of sensitivity analysis with a leave-one-out meta-analysis. The sensitivity analysis for VAS, DAS28, serum vitamin D and CRP demonstrated that removing any study did not impact the overall outcomes, but for ESR, both studies by Li et al., influence the results of the total outcomes (**Figure S1, Appendix**).

### Publication Bias

**Figure 4.**
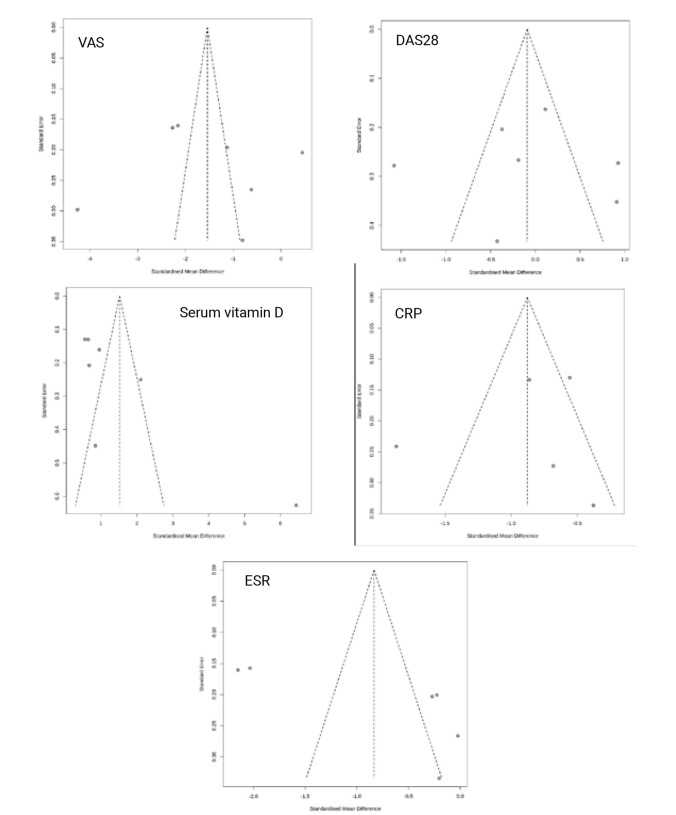
Funnel plot of RCTs of vitamin D supplementation for RA related to outcome measured.

**Figure 4** shows the funnel plot of five biomarkers and clinical outcomes in the five panels that the possibility of publication bias across the studies for some biomarkers. The results of Eger’s test showed for CRP (t=−0.04, P=0.674), DAS28 (t=−0.04, P=0.97), ESR (t=2.38, P=0.076), VAS (t=0.19, P=0.86) and Serum vitamin D (t=2.63, P=0.044). These finding revealed the asymmetry and potential publication bias for serum vitamin D but not for other indicators.

**Supplementary Figure S2.**
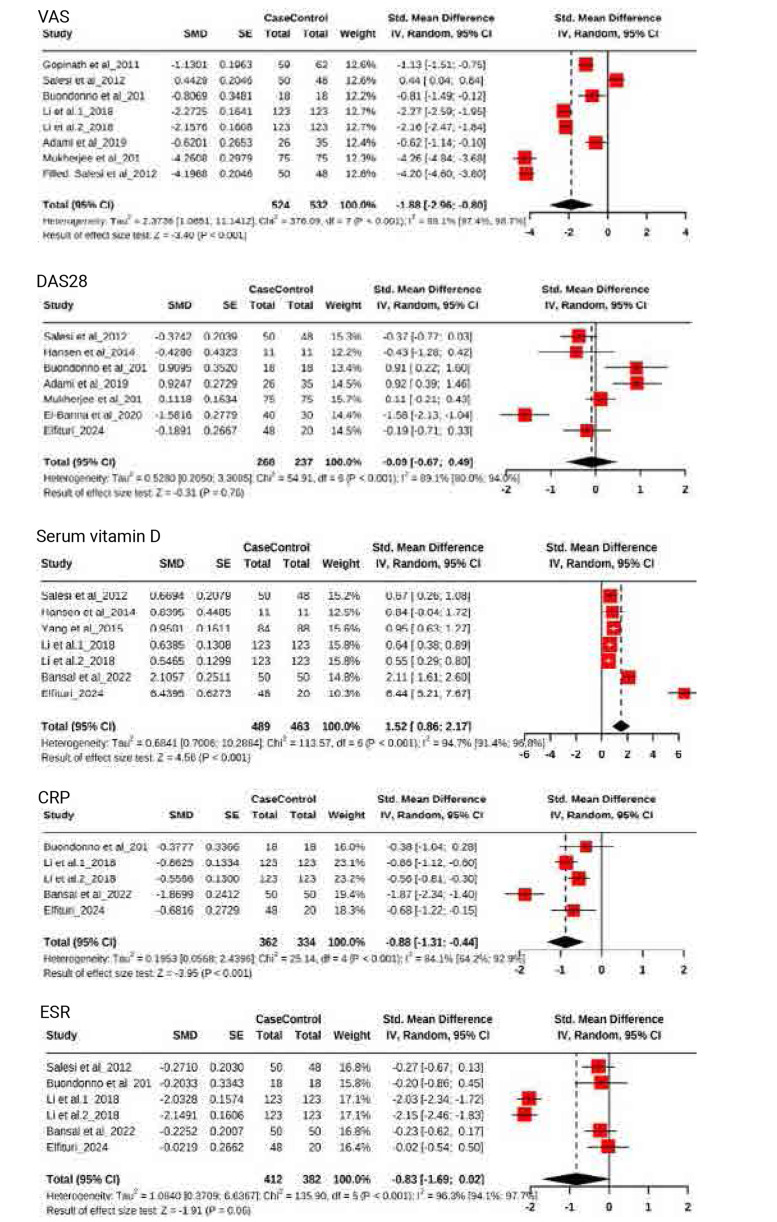
Trim & fill test. Results are expressed as Standardised mean difference (SMD) with 95%CI.

The results of the trim-and-fill test indicated that the overall effect estimate for vitamin D levels remained unchanged after adjustment. Thus, the findings suggest that publication bias had minimal impact on the results. The results of the trim and fill test are detailed in (**Figure S2, Appendix**).

## DISCUSSION

This study assessed vitamin D supplementation’s effects in RA patients, revealing significant reductions in pain (VAS) and CRP levels. However, its influence on disease activity markers like DAS28 and ESR was variable. These results align somewhat with earlier studies, though some differences persist.

Our study supports the beneficial effects of vitamin D on pain and CRP but also confirms the inconsistency of results in disease activity measures. A meta-analysis of six studies involving 438 patients showed improvements in DAS28, ESR, and TJC, but no change in CRP or PTH levels.^12^ European patients experienced more significant pain relief and increased vitamin D levels, while Asian patients showed greater reductions in TJC. Higher doses and longer treatment durations led to better outcomes.^12^

Another large meta-analysis involving 3,049 RA patients found reduced pain (VAS) and DAS28 scores, but no significant changes in CRP or ESR.^11^ Although higher vitamin D doses were associated with lower CRP in some cases, results varied widely across studies.^11^ These differences could be attributed to factors such as baseline vitamin D levels, age, and sun exposure—none of which were fully controlled for in most studies. Our findings suggest that these factors may explain why some patients respond better to supplementation than others.

In contrast to our findings in meta-analysis of RCT studies, a review of 11 studies reported reductions in DAS28, CRP, and ESR but no clear effects on HAQ scores or pain (VAS).^10,13^ This inconsistency suggests that while vitamin D may reduce certain inflammatory markers, its overall effect on pain and disability remains unclear. Variations in dosage, treatment duration, and unmeasured factors (such as diet or genetics) likely contribute to these inconsistencies, underscoring the need for more standardised research.

Variability in meta-analysis findings likely arises from differences in patient factors like disease severity, use of anti-rheumatic medications, baseline vitamin D levels, and joint damage extent. These elements can affect how well vitamin D supplementation works, leading to mixed results. Clinical benefits seem influenced by initial vitamin D status, disease duration, and structural damage severity—patients deficient in vitamin D, in early-stage RA, or with minimal joint damage generally respond better. Additionally, medication type and dose, sunlight exposure, and overall disease severity may also shape treatment outcomes.

Notably, pain originating from advanced structural lesions may be less responsive to vitamin D, as its anti-inflammatory effects primarily target cytokine-mediated pathways rather than mechanical joint damage. This may explain why disease activity measures like DAS28—which incorporate joint swelling and tenderness—were less affected in our study.

Our findings of reduced pain (VAS) and CRP support vitamin D’s anti-inflammatory role in rheumatoid arthritis. The lack of significant changes in ESR and DAS28 might stem from their differing inflammatory roles: CRP reacts quickly to acute inflammation, while ESR reflects more chronic processes influenced by various factors. Vitamin D’s greater effect on CRP suggests it primarily targets acute inflammation.^27^ Additionally, RA patients with vitamin D deficiency tend to have higher CRP levels, so supplementation in these cases can significantly lower inflammation and disease activity. Conversely, patients with adequate vitamin D levels see little change in CRP after supplementation.^28^

The DAS28 is a composite measure commonly used to assess disease activity in RA patients.^29^ It incorporates swollen and tender joint counts (SJC/TJC), ESR (or CRP), and patient-reported outcomes. While vitamin D reduced pain (VAS), it may not directly influence joint-specific measures (SJC/TJC), which are key components of DAS28 calculations. This could explain the limited effect on overall disease activity scores.^29^

The two key components of DAS28 for evaluating disease activity are CRP and ESR. Both are inflammatory markers that help quantify the level of inflammation in the body, which is important for assessing the severity of RA. CRP and ESR are crucial for determining the inflammatory state of a patient with RA and contribute to monitoring the effectiveness of treatments. Using either of these markers helps standardise DAS28 calculations across different clinical settings.^11^

This systematic review brings together current research on vitamin D supplementation for rheumatoid arthritis patients, showing notable impacts on disease activity and inflammation markers. However, the findings are limited by study quality issues, variability between trials, and signs of publication bias. Consequently, results should be interpreted carefully, and well-designed, large-scale clinical trials are needed to draw firm conclusions.

The large effect sizes reported for VAS and CRP may seem counterintuitive at first, especially given the short duration of the interventions in the study. However, such large effects have been observed in some clinical studies, particularly in inflammatory diseases like rheumatoid arthritis. This may be due to the rapid response of patients to targeted therapies or significant changes in inflammatory markers. We acknowledge that small sample sizes and publication bias can lead to imprecise estimates of effect sizes. Sensitivity analyses and trim-and-fill test results indicated that publication bias had minimal impact on the findings.

The study findings show that vitamin D supplementation significantly improves VAS and CRP but has no significant effect on DAS28 or ESR. This pattern is biologically plausible, as CRP, an acute inflammatory marker, is more sensitive to short-term changes (such as the effect of vitamin D on inflammatory pathways). In contrast, ESR and DAS28 reflect chronic disease activity and may respond better to longer-term or combined interventions.

### Future Directions for Research

To establish robust evidence for vitamin D’s clinical efficacy in RA, future research should prioritise: identification of potential confounders, implementation of rigorous control measures, standardised reporting of modifying factors, across well-designed clinical trials. These include baseline vitamin D levels, disease stage, use of concomitant medications (e.g., anti-rheumatic drugs), and demographic factors such as age and sun exposure. The observational longitudinal studies that account for these variables could provide valuable insights into which subgroups of RA patients benefit most from vitamin D supplementation. However, these observational studies suffer from confounding by indications in evaluation of treatment effects. Thus, further multicentre studies of RCT with larger sample size may explore clear evidence. Additionally, future research should explore biomarkers or clinical parameters that predict treatment response. Identifying predictive factors, such as specific inflammatory markers, genetic variations, or other immunological indicators, could help clinicians tailor vitamin D therapy to those who are most likely to benefit. Investigating the effects of varying doses and treatment durations, as well as comparing the impact of vitamin D supplementation across different ethnic groups, could also help optimise the therapeutic use of vitamin D in RA. Furthermore, research should consider the potential synergism between vitamin D and other RA treatments, which may enhance its efficacy and provide a more comprehensive approach to disease management.

## CONCLUSION

Based on our findings of meta-analysis, significant effects on the levels of serum vitamin D, VAS and CRP of patients with RA have been shown by the use of vitamin D supplements in the vitamin D supplementation groups compared to the control groups. Since the different geographic regions and durations of intervention produce different effects, further research of multicentre RCT with larger sample size is needed.

## AUTHORS’ CONTRIBUTIONS

Z.K. conceived the conception of study, designed the study protocol, searching strategy in management of data collection and analysis and wrote the first draft of the manuscript. K.H. contributed to the study design and conceptualisation, analysis and interpretation of the data, writing of the manuscript and editing and critically revising. B.H. supervised the data collection and critically revised and drafted the manuscript for intellectual content. A.S. also contributed searching strategy and meta- analysis of data and critically revised the manuscript. All authors read and approved the final manuscript.

## CONFLICTS OF INTEREST DISCLOSURES

The authors declare that the research was conducted in the absence of any commercial or financial relationships that could be construed as a potential conflict of interest.

## FUNDING

No Funding.

## ETHICS APPROVAL AND WRITTEN INFORMED CONSENTS STATEMENTS

The study protocol was approved by Ethical Board of Babol University of Medical Sciences, Babol, Iran (Ethics code: IR.MUBABOLHRI.REC.1404.036). The consent to participate was not applicable in this systematic review and meta-analysis.

## APPENDIX

### Appendix

**Supplementary Table S1.** Search strategies including the key terms and the queries for each database.

**Database**	**Key terms and the queries**
**PubMed**	(Vitamin D[Title/Abstract] OR Vitamin D Supplementation[Title/Abstract] OR 25-hydroxy-vitamin D[Title/Abstract] OR Ergocalciferols[Title/Abstract]) AND (Arthritis[Title/Abstract] OR Arthritis Rheumatoid[Title/Abstract] OR Rheumatoid Arthritis[Title/Abstract])
**Scopus**	(TITLE-ABS-KEY (Vitamin D OR (Vitamin D AND Supplementation) OR 25-hydroxy-vitamin D OR Ergocalciferols) AND TITLE-ABS-KEY (Arthritis OR Arthritis AND Rheumatoid OR (Rheumatoid AND Arthritis))
**Web of Science**	TI = (Vitamin D OR Vitamin D Supplementation OR 25-hydroxy-vitamin D OR Ergocalciferols) AND TI= (Arthritis OR Arthritis Rheumatoid OR Rheumatoid Arthritis)
**Cochrane**	(Vitamin D OR Vitamin D Supplementation OR 25-hydroxy-vitamin D OR Ergocalciferols) AND (Arthritis OR Arthritis Rheumatoid OR Rheumatoid Arthritis) in Title Abstract Keyword

## References

[1] Colotta F Jansson B Bonelli F. Modulation of inflammatory and immune responses by vitamin D. J Autoimmunity 2017;85(1):78–97. 28733125 10.1016/j.jaut.2017.07.007

[2] Heidari B Hajian-Tilaki K Babaei M. Vitamin D deficiency and rheumatoid arthritis: epidemiological, immunological, clinical and therapeutic aspects. Mediterr J Rheumatol 2019;30(2):94–102. 32185348 10.31138/mjr.30.2.94 PMC7045965

[3] Lee YH Bae SC. Vitamin D level in rheumatoid arthritis and its correlation with the disease activity: a meta-analysis. Clin Exp Rheumatol 2016;34(5):827–33. 27049238

[4] Samaan SF Taha SI Mahmoud FA Elsaadawy Y Khalil SA Gamal DM. Role of Interleukin-17 in Predicting Activity of Rheumatoid Arthritis and Systemic Lupus Erythematosus. Clin Med Insights Arthritis Musculoskelet Disord 2024;17:11795441241276880. doi: 10.1177/11795441241276880. 39351141 PMC11440548

[5] Chandrashekara S Patted A. Role of vitamin D supplementation in improving disease activity in rheumatoid arthritis: An exploratory study. Int J Rheum Dis 2017; 20(7):825–3. 26481198 10.1111/1756-185X.12770

[6] Boissier MC Assier E Falgarone G Bessis N. Shifting the imbalance from Th1/Th2 to Th17/treg: the changing rheumatoid arthritis paradigm. Joint Bone Spine 2008;75(4):373–5. 18571969 10.1016/j.jbspin.2008.04.005

[7] Ranganathan P Khalatbari S Yalavarthi S Marder W Brook R Kaplan MJ. Vitamin D deficiency, interleukin 17, and vascular function in rheumatoid arthritis. J Rheumatol 2013;40(9):1529–34. 23818717 10.3899/jrheum.130012 PMC4358878

[8] Walsh DA McWilliams DF. Pain in rheumatoid arthritis. Curr Pain Headache Rep 2012;16(6):509–17. 23109051 10.1007/s11916-012-0303-x

[9] Buondonno I Rovera G Sassi F Rigoni MM Lomater C Parisi S Vitamin D and immunomodulation in early rheumatoid arthritis: A randomized double-blind placebo-controlled study. PLoS One 2017;12(6):e0178463. 28582403 10.1371/journal.pone.0178463 PMC5459341

[10] Salesi M Farajzadegan Z. Efficacy of vitamin D in patients with active rheumatoid arthritis receiving methotrexate therapy. Rheumatol Int 2012;32:2129–33. 21523344 10.1007/s00296-011-1944-5

[11] Al-Saoodi H Kolahdooz F Andersen JR Jalili M. Effect of vitamin D on inflammatory and clinical outcomes in patients with rheumatoid arthritis: a systematic review and dose–response meta-analysis of randomized controlled trials. Nutrition Reviews 2024;82(5):600–11. 37437898 10.1093/nutrit/nuad083

[12] Guan Y Hao Y Guan Y Bu H Wang H. The Effect of Vitamin D Supplementation on Rheumatoid Arthritis Patients: A Systematic Review and Meta-Analysis. Front Med (Lausanne) 2020;30(7):596007. 10.3389/fmed.2020.596007 PMC7661491 33195358

[13] Ranjbar M Rahimlou M Fallah M Djafarian K Mohammadi H. Effects of Vitamin D Supplementation in Patients with Rheumatoid Arthritis: A Systematic Review and Meta-Analysis. Heliyon 2025;11(3):e42463. 39995929 10.1016/j.heliyon.2025.e42463 PMC11849653

[14] Moher D Liberati A Tetzlaff J Altman DG. Preferred reporting items for systematic reviews and meta-analyses: the PRISMA statement. Int J Surg 2010; 8:336–41. 20171303 10.1016/j.ijsu.2010.02.007

[15] Higgins JP Altman DG Gøtzsche PC Jüni P Moher D Oxman AD Savović J Schulz KF Weeks L Sterne JA. The Cochrane Collaboration’s tool for assessing risk of bias in randomized trials. BMJ 2011;343:d5928. 22008217 10.1136/bmj.d5928 PMC3196245

[16] Wan X Wang W Liu J Tong T. Estimating the sample mean and standard deviation from the sample size, median, range and/or interquartile range. BMC Med Res Methodol 2014;14:135. 25524443 10.1186/1471-2288-14-135 PMC4383202

[17] Hozo SP Djulbegovic B Hozo I. Estimating the mean and variance from the median, range, and the size of a sample. BMC Med Res Methodol 2005;5:13. 15840177 10.1186/1471-2288-5-13 PMC1097734

[18] Gopinath K Danda D. Supplementation of 1,25 dihydroxy vitamin D3 in patients with treatment naive early rheumatoid arthritis: a randomized controlled trial. Int J Rheum Dis 2011;14:332–9. 22004229 10.1111/j.1756-185X.2011.01684.x

[19] Yang J Liu L Zhang Q Li M Wang J. Effect of vitamin D on the recurrence rate of rheumatoid arthritis. Exp Ther Med 2015;10(5):1812–6. 26640554 10.3892/etm.2015.2747 PMC4665932

[20] Li C Yin S Yin H Cao L Zhang T Wang Y. Efficacy and safety of 22-oxa-calcitriol in patients with rheumatoid arthritis: a phase II trial. Med Sci Monit 2018;24:9127. 30554233 10.12659/MSM.911628 PMC6319165

[21] Mukherjee D Lahiry S Thakur S Chakraborty DS. Effect of 1, 25 dihydroxy vitamin D3 supplementation on pain relief in early rheumatoid arthritis. J Family Med Primary Care 2019;8(2):517–22. 30984665 10.4103/jfmpc.jfmpc_446_18 PMC6436291

[22] Bansal Y Singh P Agrawal P Saini H. Efficacy of Vitamin D Supplementation among Newly Diagnosed Cases of Rheumatoid Arthritis Proposed to be Managed by Methotrexate Monotherapy: A Randomised Controlled Study. J Clin Diagn Res 2022;16(9):RC06–RC09.

[23] Hansen KE Bartels CM Gangnon RE Jones AN Gogineni J. An evaluation of high-dose vitamin D for rheumatoid arthritis. J Clin Rheumatol 2014;20:112–4. 24561419 10.1097/RHU.0000000000000072 PMC4089514

[24] Adami G Rossini M Bogliolo L Cantatore FP Varenna M Malavolta N An exploratory study on the role of vitamin D supplementation in improving pain and disease activity in rheumatoid arthritis. Mod Rheumatol 2019;29:1059–62. 30285521 10.1080/14397595.2018.1532622

[25] El-Banna HS Gado SE. Vitamin D: does it help Tregs in active rheumatoid arthritis patients. Exp Rev Clin Immunol 2020;16(8):847–53. 10.1080/1744666X.2020.1805317 32783547

[26] Elfituri S. The effects of vitamin D supplementation on disease activity and fatigue in Libyan rheumatoid arthritis patients. Reumatologia 2024;62(2):109. 38799782 10.5114/reum/187391 PMC11114129

[27] Harrison M. Erythrocyte sedimentation rate and C-reactive protein. Aust Prescr 2015;38(3):93–4. 26648629 10.18773/austprescr.2015.034 PMC4653962

[28] Alharbi S Alharbi R Alhabib E Ghunaim R Alreefi MM Alharbi RA. Vitamin D deficiency in Saudi patients with rheumatoid arthritis. Cureus 2023;15(2): e34815. 36793500 10.7759/cureus.34815 PMC9924707

[29] Sengul I Akcay-Yalbuzdag S Ince B Goksel-Karatepe A Kaya T. Comparison of the DAS28-CRP and DAS28-ESR in patients with rheumatoid arthritis. Int J Rheum Dis 2015;18(6):640–5. 26013310 10.1111/1756-185X.12695

